# A Cross-Sectional Study of Nutrient Intake and Health Status among Older Adults in Yogyakarta Indonesia

**DOI:** 10.3390/nu9111240

**Published:** 2017-11-13

**Authors:** Tony Arjuna, Stijn Soenen, Rasita Amelia Hasnawati, Kylie Lange, Ian Chapman, Natalie D. Luscombe-Marsh

**Affiliations:** 1 Discipline of Medicine and National Health and Medical Research Council (NHMRC), Centre of Research Excellence in Translating Nutritional Science to Good Health, The University of Adelaide, Adelaide, SA 5005, Australia; stijn.soenen@adelaide.edu.au (S.S.); kylie.lange@adelaide.edu.au (K.L.); ian.chapman@adelaide.edu.au (I.C.); natalie.luscombe-marsh@csiro.au (N.D.L.-M.); 2 Department of Nutrition and Health, Faculty of Medicine, Universitas Gadjah Mada, Yogyakarta 55281, Indonesia; rasita.amelia@gmail.com; 3 Commonwealth Scientific Industrial Research Organisation (CSIRO), Nutrition and Health Program, Adelaide, SA 5005, Australia

**Keywords:** ageing, malnutrition, cognitive function, frailty, community, urban, rural

## Abstract

Many communities around the world, particularly developing countries including Indonesia, are experiencing population ageing. There is little knowledge regarding the impact of malnutrition, or its prevalence within rural compared to urban areas, on the nutritional, functional and mental status of community-living older residents in these countries. Hence, a cross-sectional study was conducted to determine socio-demographic and anthropometric characteristics, nutritional, mental and functional status, and energy and nutrient intake of community-dwelling Indonesians from both rural and urban areas of Yogyakarta. Older individuals were included in the study if they had been living in Yogyakarta for the last year and were aged ≥65 years (*n* = 527; mean ± SD age of 74 ± 7 years). Rural compared with urban participants had a lower level of education and income, more hospital admissions, less dietary protein intake, lower cognitive function, poorer nutritional status and grip strength, but faster gait speed while being more dependent on assistance to perform daily activities (all *p* < 0.05). Cognitive function was more strongly associated than nutritional status with physical function. Rural older Indonesians living in Yogyakarta were more likely than urban older people to be malnourished and cognitively impaired, and to have associated reductions in functional capacity and independence. Strategies to improve cognitive function and nutritional status are therefore important for the wellbeing of Indonesian citizens.

## 1. Introduction

Indonesia, with the 4th largest population in the world [1], is anticipated to have the greatest number of people aged 65 years or older in South East Asia within the next two decades [1]. By 2035, Indonesia’s aged population (i.e., those aged ≥ 65 years) is expected to have doubled from ~5% in 2010 to ~11% (~32 million), with the province of Yogyakarta currently having the highest proportion of older people of all 34 Indonesian provinces (i.e., ~13% compared to ~8% of the national total) [2]. Compared to the global average, the Indonesian population in general has a lower level of education, lower socio-economic status and less access to health care services [3,4,5]. The life expectancy for male and female Indonesians, respectively, is 12.2 and 14.3 years at 65 years of age, and 9.4 and 11 years at 70 years, which is substantially lower than the global average of 15.4 and 17.9 years at 65 years and 12.3 and 14.3 at 70 years [6]. Exacerbating the effects of ageing on the health and wellbeing of Indonesians are the effects of malnutrition, which for many has its onset at an early age, and impairs mental and functional status, which can in turn lead to greater rates of hospital re-admissions, greater length of hospital stay, and increased mortality [7,8,9,10,11,12]. The prevalence and effects of malnutrition, particularly on body composition and physical function, tend to be different between men and women [13,14,15]. Consequently, due to the double burden of ageing and malnutrition, it is anticipated an increased number of older adults from developing countries like Indonesia may be living in poor health, with associated low quality of life, over the next decades.

Both physiological (e.g., poor food intake) and non-physiological factors (environmental, social, psychological, polypharmacy) [8,10] play a role in the development of malnutrition, regardless of ethnicity or country of residence. However, the impact of these changes is likely be more pronounced for older adults living in the most disadvantaged communities within any country. Within Indonesia, results from limited research suggest that the prevalence of malnutrition is higher in rural compared with urban areas, due to higher poverty rates and a limited food supply, which in turn appears to reduce energy and protein intake [16]. For example, a study in West Java showed that there was higher prevalence of malnutrition in rural adults when compared with urban adults aged ≥60 years (58 rural participants: 52% at risk of malnutrition and 16% malnourished; 54 urban participants: 35% at risk of malnutrition and 2% malnourished as defined by the Mini Nutritional Assessment/MNA) and that men but not women had substantially different nutritional status [17]. In addition, a recent survey of residents of Yogyakarta indicated that those residing in rural compared with urban areas consumed significantly less protein per day (55 vs. 66 g) and had greater food insecurity (9 vs. 1 villages) [18].

Although these two studies provide insight into the prevalence of malnutrition in ageing Indonesians, and also provide some insight into the impact of a rural lifestyle on nutritional status, both studies included only small numbers of adults and did not collect socio-demographic information, or any data on functional and mental status, or biological markers associated with poor health. Moreover, many of these studies, including three other small studies that have been conducted in Indonesia [14,17,19], used screening tools that are more suited to identify nutritional status and risk among hospitalized rather than community-dwelling older people.

Given prevention, early identification and treatment of malnutrition is recognized by the World Health Organisation as a global health priority [20], the aim of this study was to determine, using a cross-sectional study design, the socio-demographic and anthropometric characteristics, and the nutritional, health, mental and functional status, of community-dwelling older men and women living in both rural and urban areas of Yogyakarta.

## 2. Materials and Methods

### 2.1. Study Design

The study had a cross-sectional design and included community-dwelling older adults living in rural or urban areas of Yogyakarta—this province is representative of Indonesia lifestyles providing a good overview with respect to the physical, demographic and socio-economic characteristics of Indonesian citizens. For instance, Yogyakarta consists of lowland and highland areas as well as pockets of slums in the the urban regions, and residents work in both modern (such as services, manufacturing/industry) and traditional (such as farming and fisheries) sectors [16]. Furthermore, residents of Yogyakarta also share the multiethnic and multiculture lifestyle of the broader regions of Indonesia; for example, the province is an education hub which has 10 public and 106 private universities, colleges and institutes that brought students and their cultures from all over the country [16]. Rural areas of Yogyakarta (and other Indoneisan regions) are characterized by villages with low population density (703 persons/km^2^), lower literacy and education levels compared to the urban areas; most rural dwellers work in agriculture [16] and low socioeconomic status—21% live below the poverty line [16,21].

### 2.2. Recruitment

The rural areas sampled for this study included those from the Kulonprogo Regency which includes ‘highland’ and Indian Ocean beach locations [16]. The urban area sampled for this study included those from the city of Yogyakarta which is located in ‘lowland’, characterized by high population density (12,699 persons/km^2^) and higher literacy and education levels compared to rural areas; most residents work in industry/manufacturing, services and wholesale/retail [16], with 9% of the residents living below the poverty line [21].

Two out of 12 sub-districts from the rural areas (i.e., Panjatan and Girimulyo) and two out of 14 sub-districts from the city of Yogyakarta (i.e., Gondokusuman and Jetis) were randomly selected using computer generated random numbers (GraphPad QuickCalcs, GraphPad Software Inc., La Jolla, CA, USA). Then, two suburbs/villages within the four sub-districts were selected as the final locations. The research team were then provided by the lead (a small group of people specially trained to assist with community health services) a list of the residents in the villages who claimed to be 65 years and older. Finally, older people were randomly selected from the lists per suburb/village; these older individuals were invited to participate in the study.

Ethical approval for the study protocol was obtained from the Medical and Health Research Ethics Committee of the Faculty of Medicine, Universitas Gadjah Mada, Indonesia (KE/FK/1177/EC/2015, 14 September 2015) and the Human Research Ethics Committee of the University of Adelaide, Australia (H-2 016-097, 28 September 2016). The study was registered on the Australian New Zealand Clinical Trial Registry (www.anzctr.org.au, Trial number ACTRN 12616000260426). Prior to data collection, the study was explained to the older individuals by the research team, including providing a volunteer information sheet. All participants provided written informed consent prior to their inclusion in the study.

### 2.3. Study Population

Sample size was calculated with GPower version 3.1.9.2 (Universität Düsseldorf, Düsseldorf, Germany). With 90% power and 5% significance level, the study requires a total sample size of 522 subjects from both the urban and rural regions to detect significant interactions in a multiple linear regression of differences in nutritional status between the two populations (effect size of 0.025).

**Figure 1:**
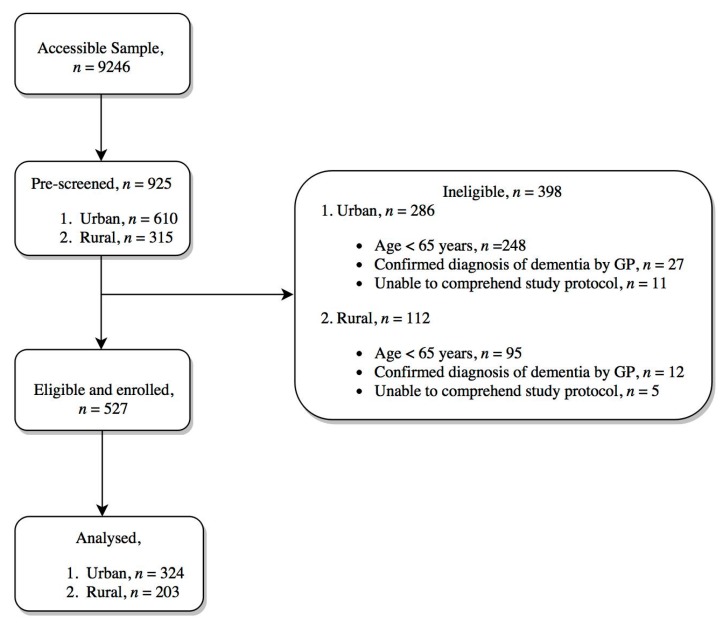
Participant recruitment process. GP: General Practitioner.

Citizens of Yogyakarta aged ≥65 years or older who had been living in the region for at least 1 year were included in the study. Ten percent (*n* = 925) of the 9246 older people living in the four sub-districts (rural area: Panjatan and Girimulyo, urban area: Gondokusuman and Jetis) were randomly selected. Older individuals were excluded if their medical records had General Practitioner confirmed diagnosis of severe dementia or cognitive impairment (10%), and/or when the study team identified individuals who were unable to comprehend the study protocol and give informed consent (4%). In addition, individuals whose age was found to be younger than 65 years based on birth records held by the civil registry were excluded from participating (86%) (Figure 1).

### 2.4. Data Collection

Data collection was conducted in the dry season (to allow safe travel to the rural areas) between September and October 2015, three months after Ramadhan (fasting month) to limit changes in food variety, energy intake and body weight (e.g., weight loss during Ramadhan and regain within a few weeks thereafter of ~1 kg) [22].

Participants were instructed to meet the research team (i.e., two investigators, twelve enumerators (graduate dietitians of Department of Nutrition and Health, Faculty of Medicine, Universitas Gadjah Mada), a nurse (with phlebotomist certification from nationally accredited CITO Pathology Laboratory, Yogyakarta, Indonesia) and cadres at their local community centre. The trained members of the research team performed the health assessments, including blood samples and nutrition and health questionnaires. All questionnaires, standard operating procedures, and participant information and informed consent forms, were translated into Bahasa Indonesia.

### 2.5. Assessments Sociodemographic Characteristics, Self-Reported Perception of Health and Medical History

Age (years) was determined by the participants’ identity card, the civil registry or voters list provided by the local government. A socio-demographic questionnaire included household information, level of education, occupation, and income. A health questionnaire included the participant’s self-description of their health, feelings of sadness or depression, requirement of help with daily activities, receiving social support when needed, and medical history of the past 6–12 months, including smoking, alcohol consumption, hospitalization (frequency of surgery, visit to a health centre or doctor, and admission and length of hospital stay—these data were cross-checked with the records from the community health centres that each participant attended or local government).

### 2.6. Anthropometric Characteristics

The following parameters were determined: body weight (kg), height (m) (Wedderburn Portable Stadiometer Model, WSHRP, Wedderburn, Auckland, New Zealand), body mass index (BMI, kg/m^2^), fat percentage, fat and lean mass (Bioelectrical impedance Analysis (BIA), Tanita Body Composition Analyzer, Model No: SC-330, Arlington Heights, IL, USA), waist, hip, mid-arm and calf circumference (cm; Seca 203 measuring tape, Hamburg, Germany), and skin-fold thickness (mm; triceps, biceps, sub-scapula, supra-iliac; Harpenden Skinfold Caliper, Model HSB-BI, British Indicators Ltd., West Sussex, UK). Measurements for all parameters were taken in duplicate and the average of the two measurment were reported; the only exception being for BIA which was done once.

### 2.7. Nutritional Status, Energy and Nutrient Intake

Nutritional status was determined by the following validated, and widely used, questionnaires: the Mini Nutritional Assessment (MNA; a score <17 indicates that the participant is malnourished, 17–23.5: at risk of malnutrition, >23.5: well nourished [23]), the Malnutrition Universal Screening Tool (MUST; a score of 0 indicates a low risk of malnutrition, 1: medium risk, ≥2: high risk [24]), the Malnutrition Screening Tool (MST; a score <2 indicates no risk of malnutrition and ≥2: risk of malnutrition [25]), the Short Nutritional Assessment Questionnaire (SNAQ; a score <2 indicates that the participant is well nourished, ≥2: moderately malnourished, ≥3: severely malnourished [26]), and the Geriatric Nutrition Risk Index (GNRI; a score <82 indicates a major risk of malnutrition, 82–91: moderate risk, 92–98: low risk, ≥98: no risk [27]). While these nutritionl screening tools have been extensively validated in Western and other Asian countries [28,29], their use in Indonesia has to date beens limited to hospital settings [30,31,32]. However, since no similar tools are available to screen the nutritional status of community living older Indonesians, the aforementioned tools were considered the most appropriate options.

Energy and nutrient intake was determined by a single 24-h recall, which provided a snapshot of usual energy and nutrient intake, and a Semi Quantitative-Food Frequency Questionnaire (SQFFQ) which has been validated among Yogyakarta residents and provides more detailed information of the food choices and source of nutrients consumed by an individual in the last 3 months [19]. Intakes of energy, macro-(protein, fat, carbohydrate) and micro-nutrients were determined by the Indonesian Food Database and Nutrisurvey (Version 2007, SEAMEO-TROPMED RCCN University of Indonesia, Jakarta, Indonesia).

### 2.8. Blood Parameters

Blood pressure and heart rate of participants were measured using Omron Blood Pressure Monitor ((Model No.: HEM-907), Omron, Kyoto, Japan). Approximately 12 mL of whole blood was collected from each participant; 4 mL for determination of Complete Blood Count (CBC) and albumin analysis, and 8 mL was converted to serum and then stored for future analysis of C-Reactive Protein and cytokines. CBC and albumin were analyzed at CITO Pathology Laboratory, the leading accredited laboratory in Yogyakarta, Indonesia.

### 2.9. Frailty, Physical and Mental, Function

Frailty was determined by the fatigue, resistance, ambulation, illness, and loss of weight questionnaire (FRAIL; a score of 0 indicates that the participant has a robust health status, 1–2: the participant is pre-frail, 3–5: frail [33]). Physical function included measurements of grip strength (kg; dominant hand, Jamar hand dynamometer, Bolingbrook, IL, USA), gait speed (m/s; 3-m walk test [34]), and the following questionnaires; Instrumental Activity of Daily Living (IADL; a score of 0 indicates that the participant is independent, 1–8: dependent [35]), physical activity (International Physical Activity Questionnaire (IPAQ) [36]). Grip strength and gait speed were measured in duplicate and average score were reported. Mental function included measurements of cognitive function, determined by the Mini Mental State Examination (MMSE; a score ≤9 indicates severe cognitive impairment, 10–19: moderate cognitive impairment, 20–24: mild cognitive impairment, 25: no cognitive impairment [37]) and depression, determined by the Geriatric Depression Scale (GDS; a score >5 indicates a suggestive depression, >9: depression [38]).

### 2.10. Statistical Analysis

Statistical analysis was performed using SPSS (Version 22.0 for Windows, IBM, New York, NY, USA). Results are presented as means and standard deviations (SD), unless stated otherwise, for all participants and a breakdown by urban and rural areas. Logistic, ordinal and multinomial regressions were used to determine the effects of location, gender and location by gender interaction on socio-demographic and healthcharacteristics. ANCOVA was used to examine the effects of location, gender and location by gender interaction on anthropometric characteristics, nutrient intake, blood parameters, frailty, physical and mental function. Spearman’s Rank test test was performed to determine between-participant associations between nutritional, frailty, physical and mental function. Lastly, based on the *priori* knowledge presented in the introduction, ANCOVA was performed to examine the independent effects of covariates (cognitive function, nutritional status and the cognitive function by nutritional status interaction) on markers of physical function; these analyses were performed without and with adjustment for location, gender and age.

Prior to analysis, data was cleaned and checked for outliers. Two member of the research team (Tony Arjuna and Rasita Amelia Hasnawati) rechecked hard copy data to clarify any identified outliers and missing values. Furthermore, skewness, kurtosis, histogram with normal curve, stem and leaf plot, and Kolomogorov-Smirnov tests were performed on numeric variables to determine normality.

## 3. Results

### 3.1. Sociodemographic Characteristics, Self-Reported Perception of Health and Medical History

Five hundred and twenty seven people aged 74 ± 7 years old (65 to 102 years) were included in the study; 203 (39%) total participants (83 men and 120 women) were from the rural area whereas 324 (61%) (132 men and 192 women) were from the urban area.

Rural compared with urban participants had a lower level of education (*p* < 0.001, Table 1), ~3.5 times lower income (*p* < 0.001) and were less likely to be retired or unemployed (56% vs. 32%, *p* < 0.001) the findings were particularly valid for the women (gender effect all *p* < 0.001). Older women were more likely to be widowed or divorced than men (61% vs. 25%, *p* < 0.001), while the older men, compared to women, were more likely to be married (74% vs. 36%, *p* < 0.001); however, these differences were attenuated after adjustment for age (adjusted *p* = 0.13). Participants from both rural and urban areas lived on average with 4 ± 2 family members.

Approximately one-quarter of the participants from both rural and urban areas rated their health as poor, or feeling sad or depressed (16–24%, Table 1). Approximately half of all study participants (40–55%) reported that they required help for more than one daily activity and rural compared with urban participants had significantly higher dependecy (49% vs. 37%, age adjusted *p* = 0.015). The majority of participants reported that they always received social support from their relatives or neighbours when needed (71–74%).

Participants from the rural, when compared with urban, areas had more hospital admissions (86% vs. 81%, *p* = 0.024, Table 1), but were less likely to have had surgery (16% vs. 35%, *p* < 0.001), particularly the women (gender effect *p* = 0.014), in the past 12 months; however, these discrepancies were diminished after adjustment for age (hospital admission age adjusted *p* = 0.12; had surgery age adjusted *p* = 0.95). Frequency of visits to the health centre (*p* = 0.55) and length of hospital stay (*p* = 0.62) was not different between the rural and urban participants.

### 3.2. Anthropometric Characteristics

Rural compared with urban participants had a lower body weight (44.4 ± 8.6 kg vs. 51.8 ± 11.5 kg, *p* < 0.001, Table 2), were not as tall (148.3 ± 8.9 cm vs. 150.4 ± 8.5 cm, *p* < 0.001) and had a lower BMI (20.1 ± 3.2 kg/m^2^ vs. 22.8 ± 4.4 kg/m^2^, *p* < 0.001). Rural compared with urban participants had a lower absolute and percentage fat mass (7.8 ± 4.4 kg vs. 12.1 ± 6.7 kg, *p* < 0.001 and, 16.7 ± 7.5% vs. 21.9 ± 9.2%, *p* < 0.001) and skinfold thickness (sum of 4 sites: 37.9 ± 18.9 mm vs. 64.1 ± 28.6 mm, *p* < 0.001). They also had a lower fat free mass (36.5 ± 5.5 kg compared to 39.7 ± 7.0 kg, *p* < 0.001) and, arm (24.0 ± 3.1 cm vs. 26.3 ± 4.1 cm, *p* < 0.001) and calf circumference (29.9 ± 4.0 cm vs. 31.7 ± 4.0 cm, *p* < 0.001). Age adjustment strengthen the gender by location effect for fat free mas (*p* = 0.019), but weakened the location effect for wasit-hip ratio (*p* = 0.057). Age adjustments on other anthropometric characteristics were non-significant.

### 3.3. Nutritional Status, Energy and Nutrient Intake

Rural compared with urban participants had poorer nutritional status according to most assessment tools (i.e., MNA: 3% vs. 6% malnourished, and 73% vs. 44% at risk of malnutrition, *p* < 0.001); MUST: 32% vs. 18% at high risk, and 17% vs. 12% at medium risk of malnutrition, *p* < 0.001); MST: 33% vs. 18% at risk of malnutrition, *p* = 0.020; GNRI: 3% vs. 2% at major risk, 22% vs. 12% at moderate risk, and 35% vs. 21% at low risk of malnutrition, *p* < 0.001). The SNAQ questionnaire however identified that nutritional status was better for rural compared with urban participants (SNAQ: 3% vs. 8% severely and 5% vs. 12% moderately malnourished, *p* = 0.001, Table 2). There was no effect of age adjustment on parameters of nutritional status.

Nutrient intake values derived from the SQFFQ methodology were not different from those derived from the 24 h recall method; hence Table 3 depicts the intakes from the 24-h recalls. Total energy, carbohydrate, fat, vitamin A, thiamine, pyridoxine, folate, vitamin E, vitamin D, magnesium, and iron intake derived from the 24-h recall were not different between the rural and urban participants. However rural compared with urban participants, had lower intakes of protein, sugar, fiber, MUFA, vitamin C, pantothenic acid, niacine, potassium, calcium, phosphorus, zinc, and higher intakes of sodium. Adjustment for age did not change the results, the only exceptions being for magnesium intake which became significantly lower among rural participants and for niacine intake which was not different between rural and urban individuals.

### 3.4. Blood Parameters

Rural compared with urban participants had higher systolic blood pressure (162 ± 27 mmHg vs. 153 ± 24 mmHg, *p* < 0.001) but slightly lower heart rates (79 ± 14 vs. 82 ± 15 beats per minute, *p* = 0.01, Table 2). Adjustment for age strengthen the gender by location effect for diastolic blood pressure (*p* = 0.048).

Rural compared with urban participants had lower values of plasma albumin concentrations (3.9 ± 0.2 vs. 4.0 ± 0.3, *p* = 0.002), hematocrit (37.4 ± 3.7% vs. 38.7 ± 4.6%, *p* = 0.001), erythrocytes (4.5 ± 0.4 vs. 4.6 ± 0.6 million cell/mL, *p* = 0.032), and lymphocytes (27.8 ± 8.0 vs. 30.9 ± 8.5%, *p* < 0.001), but higher eosinophils (4.9 ± 3.6 vs. 3.2 ± 2.6%, *p* < 0.001), neutrophils (57.9 ± 9.6 vs. 59.3 ± 9.6%, *p* = 0.022), and MCHC values (34.9 ± 1.2 vs. 34.3 ± 1.5 g/dL, *p* < 0.001). There was no effect of age adjustment on blood parameters.

### 3.5. Frailty, Physical and Mental Function

Frailty statuses were comparable among rural and urban participants (8% frail and 47% pre-frail vs. 5% frail and 52% pre-frail, *p* = 0.51, Table 2). Rural compared with urban participants had lower grip strength (15.9 ± 6.2 kg vs. 16.8 ± 6.7 kg, *p* = 0.017), but faster gait speed (0.55 ± 0.19 m/s vs. 0.51 ± 0.19 m/s, *p* = 0.050) and were more physically active (IPAQ = 910 ± 282 Metabolic Equivalent of Task (MET)-minutes/week vs. 839 ± 353 MET-minutes/week *p* = 0.010). IADL scores were greater for rural compared with urban participants indicating greater dependency for assistance (IADL score: urban, 2.7 ± 2.4 vs. rural, 3.4 ± 2.4, *p* < 0.001, Table 2) and 20% compared with 6% of urban participants, indicated they were completely dependent on assistance from others to perform IADL. However, following adjustment for age, the difference in gait speed between rural and urban paticipants was no longer significant (adjusted *p* = 0.20), while other parameters remain significantly different.

Rural compared with urban participants had lower cognitive function as assessed by the MMSE scores (7% vs. 5% had severe, 51% vs. 26% moderate, and 22% vs. 28% mild cognitive impairment, *p* < 0.001, Table 2). There was no difference in depression levels between participants from each area (GDS score: 2.8 ± 2.4 rural vs. 2.9 ± 2.4 urban, *p* = 0.72). Adjustment for age has no significant effect on parameters of mental function.

### 3.6. Correlations between Nutrient Intake with Socio-Demographic Characteristics, Nutritional Status, Physical and Mental Function

Intakes of protein, fiber, vitamins and minerals positively correlated with level of income and education (data not shown, all *p* < 0.05). Intake of potassium negatively correlated with systolic blood pressure (*r* = −0.099, *p* = 0.024). Energy and protein intakes positively correlated with body composition (fat free mass and fat mass), nutritional status (MNA, MUST, and GNRI), and both physical (grip strength, gait speed and FRAIL) and mental (MMSE and GDS) function; of these correlations, MMSE had the strongest correlation with energy and protein intakes (*r* = 0.270 and *r* = 0.288, respectively, both *p* < 0.001). Irrespective of gender, location and age, energy and protein intakes of older people with severe and moderate cognitive impairement were substantially lower than those with mild or no cognitive impairement (Energy (mean ± SEM): severe cognitive impairment 1195 ± 81 and moderate cognitive impairment 1331 ± 34 vs. mild cognitive impairment 1511 ± 39 and no cognitive impairment 1496 ± 36 kcal, *p* < 0.001; Protein (mean ± *SE*): severe cognitive impairment 34.3 ± 3 and moderate cognitive impairment 35.6 ± 1.3 vs. mild cognitive impairment 40.1 ± 1.5 and no cognitive impairment 43.6 ± 1.4 g, *p =* 0.001).

### 3.7. Correlations between Markers of Physical Function with Nutritional Status and Mental Function

**Figure 2:**
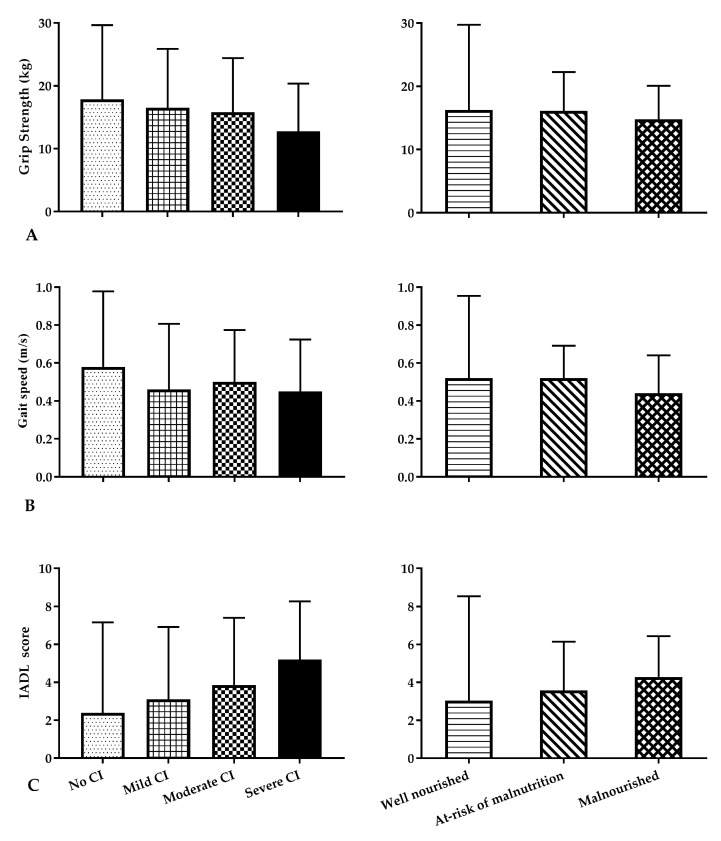
Grip strength (**A**), gait speed (**B**) and IADL (**C**) according to cognitive (left hand column) and nutritional status (right hand column) ^a^. ^a^ Data presented is age adjusted Mean ± SEM. Age adjusted *p* value for cognitive function effect adjusted for age, gender and location; *p* value for cognitive function effect (**A**) = 0.019, (**B**) = 0.019, (**C**) < 0.01; *p* value for nutritional status effect (**A**) = 0.47, (**B**) = 0.15, (**C**) = 0.09; *p* value for interaction of cognitive function by nutritional status (**A**) = 0.62, (**B**) = 0.40, (**C**) = 0.83.

The markers of physical function (grip strength, gait speed and IADL score) were associated more strongly with cognitive status as measured by MMSE, than with any other non-functional measure including nutritional status (Table 4); the correlation coefficient between MMSE score with grip strength was 0.461 (*p* < 0.001), with gait speed was 0.351 (*p* < 0.001), and with IADL was −0.440 (*p* < 0.001). There were greater increases/improvements in grip strength, gait speed and IADL as cognitive state changed from severe to moderate to mild to no impairment, compared to changes in nutritional status from malnourished to at-risk of malnutrition to well nourished (Figure 2). The majority of participants classified as having severe to mild cognitive impairment were at risk of malnutrition (severe: 21/30, moderate: 121/187 and mild: 85/134, respectively), while the majority (107/176) of participants with no cognitive impairment were well nourished (*p* < 0.001).

## 4. Discussion

This is the first study to our knowledge that has comprehesively evaluated and compared the socio-demographic and anthropometric characteristics, nutritional, cognitive and functional status, energy and nutrient intake of community-dwelling older men and women in rural and urban areas of Indonesia. The rural compared to urban participants in this study had lower levels of education and income, less dietary protein intake, more hospital admissions in the previous 6 months, lower cognitive function, poorer nutritional status (including lower body weight, height, BMI, arm and calf circumference, skinfold thickness, fat percentage, fat and fat free mass and plasma albumin concentrations), and reduced grip strength. Although they had significantly faster gait speeds, they rated themselves as being more dependent on assistance from others to perform instrumental activities of daily living including shopping, food preparation, housekeeping, transport, managing medications and finances. Cognitive function was the measure that best correlated with the measured functional outcomes of grip strength, gait speed and activities of daily living, and more strongly associated with these than any measure of nutritional status in this study.

The prevalence of undernutrition (both malnutrition and at risk of malnutrition) as assessed by MNA among community-dwelling older people in this study was comparable to findings from other developing countries such as India and Iran [39,40,41], but significantly higher than those from more developed countries such as Japan, Taiwan, Poland and France [42,43,44,45]. A French study of 692 rural- and 8691 urban-based older people reported that undernutrition was more prevalent in the urban than rural areas (7.4% vs. 18.5%) [45], while a study conducted in Poland reported that the mean MNA scores of nursing home residents (*n* = 879) were significantly lower (indicating worse nutrition) than those of either urban (*n* = 1003) or rural (*n* = 890), community-living residents. Notably, the mean MNA score of our urban and rural Indonesian community-living residents (i.e., 24.5 ± 3.5 vs. rural 23.3 ± 3.9) were comparable to the values of the Polish nursing home residents (21.3 ± 4.8) [44].

SNAQ was the only questionnaire used in this study that indicated a higher prevalence of severe and moderate malnutrition among urban than rural participants (8% vs. 3% severely malnourished, and 12% vs. 5% moderately malnourished). A closer look at each items of SNAQ questionnaire revealed that more of the study participants from the urban areas reported losing more than 6 kg which gave them 2 points and classified them as moderately malnourished (*n* = 4 vs. 1, equals to 2 vs. 0.5% of rural population). Additionally, there were more participants who reported taking supplemental drinks from the urban than rural area (*n* = 10 vs. 0, equals to 5 vs. 0% of rural population), which may further inflate the SNAQ score as supplemental drinks are more widely available to those living in urban than rural areas. Energy and protein intakes of Indonesian participants in this study (energy: 1411 ± 39 kcal and protein: 39.5 ± 17.6 g) were representative of the broader Indonesian population; for example, in the recent large “Total Diet Study” which involved 145,360 Indonesians from the 34 provinces, energy intake of people aged 55 years or more living in rural areas was 1615 ± 632 kcal for men and 1301 ± 509 kcal for women, whereas in urban areas they were 1676 ± 641 kcal for men and 1332 ± 516 kcal for women [46]. Rural participants in the present study had substantially lower protein in their diet than urban participants (by ~6 ± 17 g per day), and this intake was negatively associated with income. This lower protein intake may have resulted, at least in part, in the lower nutritional status and lower body fat free mass observed in the rural participants. This finding also suggests that food security and supply are substanital issues that affect the health and nutritional status of rural older adults. Hence, improving food availability and affordability in rural areas should be a focus for the Indonesian government.

Rural participants, especially women, had higher systolic blood pressures than urban participants, and may therefore, be at greater cardiovascular risk than urban older people. The reasons for the higher systolic blood pressures in rural participants are not clear. Dietary factors may be partly responsible; for example, rural participants reported lower potassium and higher sodium intakes and both factors are associated with blood pressure increases [47,48]. In addition, the higher blood eosinophil counts for rural participants may be caused by a higher prevalence of soil-transmitted helminth parasite infections in farmers. The prevalence of worm infections (e.g., ascaris lumbricoides, hookworm, trichuris trichura, and enterobius vermicularis) is usually higher in rural than urban areas (52–86% compared to 28%) [49,50].

Grip strength values of our rural and urban participants from Yogyakarta (15.9 ± 6.2 and 16.8 ± 6.7 kg) were comparable to those reported from other Asian countries and Hispanic-American communities; for example, in Singapore: 362 rural and urban adults aged ≥65 years had a grip strength of 31.2 ± 9.2 and 24.4 ± 8.5 kg [51]; in Taiwan: 558 adults aged ≥75 years had a grip strength of 22.3 ± 6.2 kg [52]; and in Hispanic-America: 2381 adults aged ≥65 years had a grip strength of 23.3 ± 9.1 kg [53]. In contrast, grip strength of the Indonesian participants in this study were substantially lower than community-living adults aged 65 plus years from Western countries including Belgium, Israel, Spain, UK and USA [54,55,56,57]. In fact, the mean grip strength observed for the Yogyakarta participants in this study, fell within the values that have been reported for western people aged 90 years or more (11.5 ± 5.6 kg for women and 19.5 ± 8.2 kg for men) [57]. The range of gait speeds observed in this study for the rural and urban participants (i.e., 0.34 to 0.72 m/s) is comparable to values previously reported for Hispanic-American older adults, but is substantially slower than for predominatly Caucasian and Afro-American older community-living adults (0.70 to 1.42 m/s), and comparable to institutionalized western people aged 90 years or more (0.49 ± 0.21 m/s for 90 years, and 0.43 ± 0.19 m/s for 95+ years) [58,59].

Substantially lower gait speeds and grip strengths of many populations, including in Indonesia, may be related to a smaller body frame, smaller stride length, and more relaxed pace of life, particulalry given that body size and weight are positively associated with both markers of physial function [60,61]. Indeed, the participants in this study had lower mean heights, weights and BMIs than “Western” populations of the same age. Therefore, the clinical significance of the lower grip strength and gait speed within some populations compared with others remains unclear, and it is highly likely that different cut offs indicating higher risk of malnutrition, frailty and impaired physical and mental function need to be determined for specific enthnicities.

This study is the first to our knowledge to report the level and prevalence of cognitive impairment among Indonesians from rural compared with urban areas of Yogyakarta. The lower level of cognitive funcioning observed in the rural participants is probably best explained by differenences in their level of education, but our current findings also indicate lower cognitive function may be related to their poorer nutritional status. Cognitive function appeared to be the strongest predictor of reported energy and protein intake. Previous study of 449 community-living Korean aged ≥60 years reported that good cognitive function as assessed by MMSE was significantly and positively associated with energy and protein intake [62]. Similarly, a study of 178 Spanish adults aged ≥65 years showed that individuals that had no cognitive impairment compared to those with mild cognitive impairment had a higher energy intake by an average 122 kcal/day [63]. An Italian based study of 1651 adults with mean age of 70 years also reported that as cognitive function decreased from ‘no’ to ‘severe’ impairment, daily energy intake tended to decrease by an average of 3.3 MJ/day; moreover, the reduction was more marked for men than women [64]. Although causality cannot be determined, the data suggest that older Indonesian with an increased level of impaired cognitive function are likely to be malnourished. Hence, a malnutrition prevention program, particularly targeting older people with cognitive impairment needs more emphasis on involving other family members, considering the strong family bond and care dependancy among Indonesian elderly [65].

Our findings regarding the relationship between cognitive function and markers of physical function are consistent with previously reported associations from other countries [53,66,67]. In a prospective study of older adults from the United States, reduced grip strength was associated with the presence of persistent, mild, cognitive impairment (hazard ratio (*HR*): 1.34, 95% confidence interval (CI) 1.02–1.75), while the risk of developing mild cognitive impairment was associated with both reduced grip strength (*HR*: 1.28, 95% CI 1.07–1.54) and gait speed (*HR*: 1.27, 95% CI 1.11–1.45) [66]. In a separate prospective study from the United States, reduced cognitive function with ageing was associated with decreased grip strength [53,67], and older people who developed Alzheimer’s disease had lower grip strengths than those who did not [53,67]. Our findings now extend these to a developing country, suggesting that the relationship between cognition and markers of functional status is an age-related phenomenon evident in older populations across different regions of the world. Findings of this study also confirm results from a systematic review which showed that association between cognitive function and markers of physical function were found in both cross-sectional and longitudinal studies, implying that cognitive function also predicts future decline in physical function [68].

The importance of the functional parameters, particularly gait speed and grip strength, that we measured, is their assocation in numerous studies with important clinical outcomes such as quality of life, independence, frailty, hospital admissions and survival [55,56,57,58,66,69,70]. In recognition of the importance of these funcational measures, they are now included in most criteria for the definition of sarcopenia [71]. These functional measures are therefore important measurements of health and wellbeing of older populations, and our findings support the importance of assessing cognitive function also.

A limitation of the study was that the cross-sectional design does not allow determination of causal relationships between the nutritional, physical and mental function of older people living in rural and urban areas of Indonesia. Nevertheless, this study was among the first to use multiple tools to assess nutritional status, physical and mental function, and to identify Indonesian specific values for each range of these tools. However, our findings also highlight the fact that current scoring sytems within the tools used to classify frailty and sarcopenia—tools that have largely been developed in ‘Western’ population [71]—may not be applicable and thus there need to be population specific criteria. This is a justified concern since urban-residing Indonesians are more aware of their health, and hence, more likely to have routine health check-ups by a General Practitioners, which in turn may lead to earlier diagnosis of disease. However, we speculate the associations found between cognitive impairment and nutritional and functional parameters may have been stronger with inclusion of more individuals with severe cognitive impairment and also with the use of more sensitive tools than the MMSE to measure subtle changes in a range of cognitive abilities [72,73].

## 5. Conclusions

In conclusion, rural, when compared to urban, older Indonesians living in Yogyakarta were more likely to be malnourished and cognitively impaired, which were associated with reduced functional capacities, and more dependent for help. Strategies to increase both health-professional and public awareness of the nutritional and cognitive issues facing older Indonesians, and the development of targeted intreventions to improve cognitive function and nutritional status, are therefore important for the health and wellbeing of older Indonesians.

## Figures and Tables

**Table 1:** Sociodemographic characteristics, self-reported perception of health and medical history of the rural and urban study participants ^a^.

Characteristics	Urban		Rural		Location Effect	Gender Effect	Interaction Effect Location by Gender
	Male (*n* = 132)	Female (*n* = 192)	Male (*n* = 83)	Female (*n* = 120)			
**Socio-Demographic**							
Age ^b^	74.7 ± 6.4	74.3 ± 6.8	72.8 ± 7.1	73.8 ± 7.9	0.06	0.67	0.31
Marital Status ^c^							
1. Married	91 (69)	68 (35)	68 (82)	44 (37)	0.78	<0.001 ^k^	0.11
2. Widowed/Divorced	40 (30)	119 (62)	14 (17)	72 (60)			
3. Single/Never Married	1 (1)	5 (3)	1 (1)	4 (3)			
Last Education ^d^					<0.001	<0.001	0.06
1. Uneducated	21 (16)	79 (41)	31 (37)	93 (77)			
2. Elementary School	34 (26)	23 (12)	35 (42)	24 (20)			
3. Junior High School	22 (16)	35 (18)	8 (10)	1 (1)			
4. Senior High School	30 (23)	35 (18)	8 (10)	2 (2)			
5. University/Academy	25 (19)	20 (11)	1 (1)	0 (0)			
Occupation ^e,f^					<0.001 ^l^	<0.001 ^l^	0.38
1. Farmer/breeder/fisherman	1 (1)	2 (1)	45 (54)	40 (33)			
2. Labor/farming labor	10 (8)	3 (2)	19 (23)	24 (20)			
3. Private employee/civil servant/military/entrepreneur	21 (15)	33 (17)	3 (3)	3 (2)			
4. Other works	50 (38)	22 (11)	2 (3)	2 (2)			
5. Unemployed/Retired	50 (38)	132 (69)	14 (17)	51 (43)			
Monthly income group ^d,g^					<0.001	0.57	0.61
1. Low	68 (52)	122 (64)	75 (91)	111 (92)			
2. Middle	23 (17)	38 (20)	2 (2)	4 (3)			
3. High	19 (14)	14 (7)	4 (5)	2 (2)			
4. Very high	22 (17)	18 (9)	2 (2)	3 (3)			
Poverty ^c,h^					<0.001	0.95	0.68
1. Poor	43 (33)	71 (37)	64 (77)	93 (77)			
2. Non-poor	89 (67)	121 (63)	19 (23)	27 (23)			
Smoking Status ^c,i^					0.21	0.003	0.53
1. Yes	34 (26)	1 (0)	38 (46)	3 (2)			
2. No	52 (39)	188 (98)	20 (24)	117 (98)			
3. Smoke in the past	46 (35)	3 (2)	25 (30)	0 (0)			
Alcoholic Drink Consumption ^c,j^					0.94	0.99	0.99
1. Yes	3 (2)	3 (2)	0 (0)	2 (2)			
2. No	129 (98)	189 (98)	83 (100)	118 (98)			
**Health and Medical History**							
Self-description of health ^d^					0.32	0.31	0.342
1. Excellent	71 (54)	105 (55)	57 (68)	73 (61)			
2. Fair	29 (22)	44 (23)	13 (16)	26 (22)			
3. Poor	32 (24)	43 (22)	13 (16)	21 (17)			
Feel sad or depressed ^c^					0.25	0.15	0.60
1. Yes	21 (16)	45 (23)	13 (16)	20 (17)			
2. No	111 (64)	147 (77)	70 (84)	100 (83)			
Requiring help for daily activities ^d^					0.08	0.56	0.97
1. 0–1 activity	80 (60)	123 (64)	37 (45)	66 (55)			
2. 2–4 activities	30 (23)	43 (22)	37 (45)	32 (27)			
3. 5–8 activities	22 (17)	26 (14)	9 (10)	22 (18)			
Receiving social support when needed in the past year ^d^					0.46	0.96	0.99
1. Always	93 (71)	137 (71)	61 (74)	89 (74)			
2. Sometimes	23 (17)	29 (15)	16 (19)	21 (18)			
3. Never	16 (12)	26 (14)	6 (7)	10 (8)			
Hospital admission in the past year ^d^					0.024	0.99	0.14
1. 0 admission	17 (13)	45 (23)	11 (13)	17 (14)			
2. 1–2 admissions	98 (74)	130 (68)	61 (74)	86 (72)			
3. >2 admissions	17 (13)	17 (9)	11 (13)	17 (14)			
Had surgery in the past year ^c^					<0.001	0.014	0.12
1. Yes	51 (39 )	63 (33)	20 (24)	13 (11)			
2. No	81 (61)	129 (67)	63 (76)	107 (89)			
Frequency of visit to Health Centre or Doctor in the past 6 months ^d^					0.55	0.25	0.90
1. 0 visit	52 (39)	68 (36)	35 (42)	45 (37)			
2. 1–5 visits	48 (36)	77 (40)	36 (44)	56 (47)			
3. 6–10 visits	26 (20)	37 (19)	11 (13)	16 (13)			
4. >10 visits	6 (5)	10 (5)	1 (1)	3 (3)			
Length of hospital stay in the past 6 months (days) ^d^					0.62	0.33	0.53
1. 0 day	120 (91)	177 (92)	80 (96)	114 (95)			
2. 1–5 days	4 (3)	4 (2)	3 (4)	2 (2)			
3. 6–10 days	6 (5)	8 (4)	0 (0)	4 (3)			
4. >10 days	2 (1)	3 (2)	0 (0)	0 (0)			

^a^ Data represent N (%); ^b^ Data presented is unadjusted Mean ± Standard deviation; Regression analysis to determine the effects of location, gender and location by gender interaction with ^c^ Logistic regression; ^d^ Ordinal regression; ^e^ Multinomial regression; ^f^ Based on occupation category set by Ministry of Health and Indonesian Bureau of Statistics; ^g^ Monthly income category set by Indonesian Bureau of Statistics where Income of <Rp.1,500,000 = low, Rp.1,500,000 to Rp.2,500,000 = middle, Rp.2,500,000 to 3,500,000 = high, and ≥Rp.3,500,000 = very high; ^h^ Poverty line as defined by Indonesian Bureau of Statistics where income of <Rp.600,000 = poor and ≥Rp.600,000 = non poor; ^i^ Smoking status, yes = actively smoking at least 1 cigarette/day, no = non active smoker and never smoked in the past, smoke in the past = non active smoker but smoked in the past; ^j^ Alcoholic drink consumption, yes = consuming at least 1 standard drink/week, no = consuming less than 1 standard drink/week or never consumed alcoholic drink; ^k^ Married was reference category, significant difference with widowed; ^l^ Unemployed/retired is reference category, significant difference to Farmer/breeder/fisherman and Labor/farming labor. *p* values were not adjusted for age, results for all age adjusted effects of location, gender and location by gender are described in the text.

**Table 2:** Anthropometric characteristics, nutritional, functional and mental status, and blood parameters ^a,b^.

Characteristics	Urban		Rural		Location Effect	Gender Effect	Interaction Effect Location by Gender
	Men (*n* = 132)	Women (*n* = 192)	Men (*n* = 83)	Women (*n* = 120)			
**Anthropometry and Body Composition**							
Weight (kg)	55.3 ± 11.1	49.4 ± 11.2	47.5 ± 8.5	42.2 ± 8.0	<0.001	<0.001	0.76
Height (cm)	157.4 ± 6.0	145.6 ± 6.4	154.7 ± 7.5	143.8 ± 6.8	<0.001	<0.001	0.44
BMI (kg/m^2^) ^c^	22.2 ± 3.8	23.2 ± 4.7	19.8 ± 2.7	20.4 ± 3.5	<0.001	0.025	0.61
Fat percentage (%) ^d^	18.4 ± 6.0	24.4 ± 10.3	14.3 ± 4.5	18.4 ± 8.6	<0.001	<0.001	0.20
Fat mass (kg) ^d^	10.7 ± 5.0	13.1 ± 7.5	7.0 ± 3.2	8.4 ± 5.1	<0.001	0.001	0.34
Fat free mass (kg) ^d^	44.7 ± 6.9	36.3 ± 4.7	40.3 ± 5.4	33.8 ± 3.8	<0.001	<0.001	0.049
Waist circumference (cm)	82.1 ± 11.5	78.6 ± 12.0	73.1 ± 7.4	71.8 ± 8.3	<0.001	0.012	0.25
Hip circumference (cm)	92.1 ± 7.8	92.9 ± 10.5	84.9 ± 5.9	84.7 ± 7.1	<0.001	0.68	0.51
Waist—hip ratio	0.9 ± 0.1	0.8 ± 0.1	0.9 ± 0.1	0.8 ± 0.1	0.045	0.002	0.14
Arm circumference (cm)	26.6 ± 3.5	26.2 ± 4.5	24.2 ± 2.7	23.8 ± 3.3	<0.001	0.22	0.99
Calf circumference (cm)	32.4 ± 3.7	31.3 ± 4.1	30.4 ± 3.0	29.5 ± 4.5	<0.001	0.005	0.76
Skinfold thickness (mm)	55.7 ± 25.6	69.9 ± 29.2	31.1 ± 15.4	42.6 ± 19.7	<0.001	<0.001	0.53
1. Biceps	9.0 ± 8.7	12.5 ± 7.6	4.4 ± 2.6	6.9 ± 7.9	<0.001	<0.001	0.46
2. Triceps	13.1 ± 8.7	18.9 ± 9.7	7.3 ± 4.9	12.0 ± 5.7	<0.001	<0.001	0.43
3. Sub-scapula	17.2 ± 8.8	19.2 ± 9.6	10.6 ± 4.9	12.6 ± 6.1	<0.001	0.007	0.99
4. Supra-iliac	16.4 ± 9.8	19.4 ± 8.4	8.7 ± 4.9	11.1 ± 5.8	<0.001	<0.001	0.63
**Nutritional Status**							
MNA ^e^	23.2 ± 3.4	22.9 ± 3.5	21.6 ± 2.4	21.9 ± 2.7	<0.001	0.97	0.39
MUST ^f,g^	0.6 ± 0.9	0.5 ± 0.9	1.0 ± 1.1	0.8 ± 1.0	<0.001	0.54	0.47
MST ^f,h^	0.5 ± 0.9	0.6 ± 0.8	0.9 ± 1.0	0.7 ± 1.0	0.020	0.29	0.28
SNAQ ^f,i^	0.5 ± 1.0	0.6 ± 1.1	0.3 ± 0.8	0.2 ± 0.7	0.001	0.76	0.22
GNRI ^j^	98.9 ± 6.3	98.7 ± 6.3	95.2 ± 5.9	95.9 ± 6.9	<0.001	0.60	0.30
**Blood Parameters**							
Systolic blood pressure (mmHg)	150 ± 23	154 ± 24	155 ± 24	167 ± 27	<0.001	0.001	0.058
Diastolic blood pressure (mmHg)	82 ± 15	83 ± 15	79 ± 16	85 ± 15	0.63	0.023	0.055
Heart rate (beats/minute)	79 ± 14	84 ± 15	74 ± 14	82 ± 13	0.010	<0.001	0.37
Haemoglobin (g/dL)	14.0 ± 1.7	12.8 ± 1.5	13.7 ± 1.3	12.6 ± 1.4	0.09	<0.001	0.56
Haematocrit (%)	40.3 ± 4.5	37.6 ± 4.3	38.9 ± 3.4	36.4 ± 3.5	<0.001	<0.001	0.68
Erythrocytes (million cell/mL)	4.7 ± 0.6	4.5 ± 0.5	4.6 ± 0.4	4.4 ± 0.4	0.019	<0.001	0.33
Thrombocytes (thousand/mL)	255.2 ± 65.1	286.4 ± 73.0	258.1 ± 69.9	287.2 ± 79.3	0.78	<0.001	0.88
Leucocytes (thousand cell/mL)	6.8 ± 1.8	7.4 ± 2.1	6.6 ± 1.6	7.0 ± 1.7	0.10	0.003	0.64
Eosinophils (%)	4.1 ± 3.1	2.7 ± 2.0	4.7 ± 3.3	5.0 ± 3.8	<0.001	0.04	0.001
Basophils (%)	0.3 ± 0.4	0.1 ± 0.3	0.2 ± 0.4	0.1 ± 0.3	0.16	<0.001	0.16
Neutrophils (%)	55.4 ± 9.6	59.5 ± 9.2	60.3 ± 9.3	58.6 ± 9.8	0.022	0.18	0.001
Lymphocytes (%)	32.0 ± 9.0	30.2 ± 8.1	26.3 ± 7.5	28.8 ± 8.1	<0.001	0.67	0.003
Monocytes (%)	8.1 ± 2.3	7.5 ± 2.3	8.5 ± 2.5	7.5 ± 2.6	0.39	<0.001	0.33
MCV (fl)	85.3 ± 5.8	84.2 ± 5.9	84.7 ± 4.8	82.9 ± 6.8	0.09	0.008	0.51
MCH (pg)	29.6 ± 2.0	28.7 ± 2.1	29.9 ± 2.0	28.8 ± 2.8	0.30	<0.001	0.84
MCHC (g/dL)	34.8 ± 1.6	34.0 ± 1.4	35.3 ± 1.0	34.6 ± 1.2	<0.001	<0.001	0.67
RDW (%)	14.2 ± 1.2	14.3 ± 1.8	14.3 ± 1.3	14.4 ± 1.6	0.58	0.40	0.86
Albumin (g/dL)	4.1 ± 0.3	4.0 ± 0.2	3.9 ± 0.2	4.0 ± 0.2	0.001	0.75	0.13
**Frailty and Physical Function**							
FRAIL ^f,k^	0.7 ± 0.8	1.0 ± 0.9	0.7 ± 0.9	0.9 ± 1.0	0.51	0.14	0.38
Grip strength (kg)	21.4 ± 7.1	13.7 ± 4.2	18.9 ± 6.4	13.7 ± 5.0	0.017	<0.001	0.013
Gait Speed (m/s) ^d^	0.55 ± 0.22	0.49 ± 0.18	0.59 ± 0.19	0.51 ± 0.19	0.050	<0.001	0.517
IADL ^l^	3.0 ± 2.4	2.6 ± 2.4	4.1 ± 2.2	3.0 ± 2.4	<0.001	0.001	0.09
IPAQ (MET-minutes/week) ^m^	819.5 ± 321.7	852.4 ± 374	929.9 ± 266.3	896.1 ± 292.7	0.010	0.98	0.27
**Mental Function**							
MMSE ^n^	22.3 ± 5.8	21.4 ± 6.7	20.5 ± 6.2	16.3 ± 6.2	<0.001	<0.001	0.004
GDS ^o^	2.9 ± 2.6	2.9 ± 2.3	2.8 ± 2.5	2.9 ± 2.4	0.72	0.89	0.73

^a^ ANCOVA test to determine the effects of location, gender and location by gender interaction; ^b^ Data presented is unadjusted Mean ± Standard deviation; ^c^ BMI: Body Mass Index; ^d^ Missing data from 10 participants due to unable to stand firmly on the BIA machine and perform 3-m walk test; ^e^ MNA: Mini Nutritional Assessment, <17 = malnourished, 17–23.5 = at risk of malnutrition, >23.5 = well nourished; ^f^ Ordinal regression test to determine the effects of location, gender and location by gender interaction; ^g^ MUST: Malnutrition Universal Screening Tool, 0 = low risk, 1 = medium risk, 2 or more = high risk; ^h^ MST: Malnutrition Screening Tool, ≥2 = risk of malnutrition; ^i^ SNAQ: Short Nutritional Assessment Questionnaire, <2 = well nourished, ≥2 = moderately malnourished; ≥3 = severely malnourished; ^j^ GNRI: Geriatric Nutrition Risk Index, >98 = no risk, 92 to ≤98 = moderate risk, 82 to <92 = low risk, <82 = major risk; ^k^ FRAIL: Fatigue Resistance Ambulation Illnesses and Loss of weight, 0 = robust health status, 1–2 = pre-frail, 3–5 = frail; ^l^ IADL: Instrumental Activities of Daily Living, 0 = independent, 1–8 = dependent; ^m^ IPAQ: International Physical Activity Questionnaire, MET: Metabolic Equivalent of Task; ^n^ MMSE: Mini Mental State Examination scale, ≥25 = no cognitive impairment, 20–24 = mild cognitive impairment, 10–19 moderate cognitive impairment, ≤9 = severe cognitive impairment; ^o^ GDS: Geriatric Depression Scale, >5 = suggestive of depression, >9 = depression. *p* values were not adjusted for age, results for all age adjusted effects of location, gender and location by gender are described in the text.

**Table 3:** 24-h recall nutrient intakes ^a,b^.

Nutrients	Urban		Rural		Location Effect	Gender Effect	Interaction Effect Location by Gender
	Male (*n* = 132)	Female (*n* = 192)	Male (*n* = 83)	Female (*n* = 120)			
Energy (kcal)	1530 ± 500	1365 ± 445	1520 ± 447	1278 ± 402	0.24	<0.001	0.35
Protein (g)	45 ± 21	40 ± 18	39 ± 15	34 ± 13	<0.001	0.003	0.91
Carbohydrate (g)	229 ± 72	197 ± 64	237 ± 75.0	186 ± 60	0.77	<0.001	0.14
Dietary fibre (g)	1 ± 24	9 ± 10	8 ± 5	7 ± 5	0.006	0.18	0.34
Sugar (g)	35 ± 18	31 ± 19	30 ± 20	23 ± 16	<0.001	0.002	0.25
Fat (g)	51 ± 24	48 ± 22	48 ± 21	46 ± 22	0.13	0.28	0.72
PUFA (g) ^c^	6 ± 4	6 ± 4	6 ± 5	6 ± 6	0.62	0.48	0.15
MUFA (g) ^d^	9 ± 6	8 ± 5	7 ± 4	8 ± 7	0.040	0.92	0.11
Saturated Fat (g)	31 ± 22	29.6 ± 29.3	28.3 ± 12.9	26 ± 12	0.17	0.45	0.83
Cholesterol (mg)	117 ± 146	106 ± 136	123 ± 163	94 ± 147	0.83	0.13	0.51
Vitamin A (µg) ^e^	1391 ± 866	1350 ± 1005	1575 ± 1127	1364 ± 780	0.25	0.14	0.32
Thiamine (mg)	0.5 ± 0.2	0.5 ± 0.3	0.5 ± 0.2	0.5 ± 0.2	0.81	0.049	0.26
Riboflavin (mg)	0.4 ± 0.3	0.4 ± 0.3	0.4 ± 0.3	0.4 ± 0.3	0.08	0.49	0.82
Pantothenic acid (mg)	1.6 ± 0.9	1.5 ± 0.9	1.3 ± 0.8	1.2 ± 0.7	0.001	0.17	0.99
Pyridoxine (mg)	0.7 ± 0.4	0.7 ± 0.4	0.7 ± 0.4	0.6 ± 0.4	0.83	0.19	0.58
Folate (µg) ^f^	118 ± 76	119 ± 78	129 ± 145	108 ± 64	0.97	0.20	0.15
Vitamin C (mg)	41 ± 54	47 ± 46	34 ± 29	36 ± 29	0.016	0.34	0.59
Vitamin D (µg)	1.1 ± 2.8	1.0 ± 2.6	1.0 ± 2.3	0.9 ± 2.0	0.87	0.57	0.98
Vitamin E (mg) ^g^	3.4 ± 3.6	3.1 ± 2.7	3.4 ± 3.5	3.7 ± 4.5	0.45	0.99	0.33
Sodium (mg)	1241 ± 566	1308 ± 619	1756 ± 888	1639 ± 722	<0.001	0.69	0.14
Potassium (mg)	1231 ± 601	1237 ± 585	1108 ± 508	1045 ± 469	0.002	0.62	0.53
Calcium (mg)	352 ± 244	346 ± 207	309 ± 135	301 ± 181	0.017	0.70	0.95
Magnesium (mg)	168 ± 122	144 ± 75	135 ± 66	142 ± 87	0.36	0.29	0.06
Phosphorus (mg)	562 ± 283	517 ± 238	466 ± 171	444 ± 190	<0.001	0.11	0.58
Iron (mg)	8.2 ± 4.2	7.5 ± 3.8	8.7 ± 10.3	7.4 ± 3.6	0.71	0.06	0.52
Zinc (mg)	3.6 ± 2.0	3.3 ± 1.7	3.0 ± 2.5	2.7 ± 1.3	0.001	0.10	0.95

^a^ ANCOVA test to determine the effects of location, gender and location by gender interaction; ^b^ Data presented is unadjusted Mean ± Standard deviation; ^c^ PUFA = Polyunsaturated fatty acid; ^d^ MUFA = Monounsaturated fatty acid; ^e^ Vitamin A = retinol equivalents; ^f^ Folate = total folic acid; ^g^ Vitamin E = tocopherol equivalent. *p* values were not adjusted for age, results for all age adjusted effects of location, gender and location by gender are described in the text.

**Table 4:** Spearman’s rank test between parameters of nutritional status, physical and mental function ^a^.

	Energy	Protein	MNA	MUST	MST	SNAQ	GNRI	MMSE	GDS	Grip Strength	Gait Speed	IADL
**Protein**	0.840 **											
**MNA**	0.208 **	0.230 **										
**MUST**	−0.097 *	−0.121 **	−0.636 **									
**MST**	−0.074	−0.075	−0.474 **	0.208 **								
**SNAQ**	0.005	0.018	−0.288 **	0.191 **	0.466 **							
**GNRI**	0.082	0.111 *	0.645 **	−0.722 **	−0.168 **	−0.110 *						
**MMSE**	0.270 **	0.288 **	0.383 **	−0.207 **	−0.138 **	0.022	0.280 **					
**GDS**	−0.074	−0.091 *	−0.356 **	0.107 *	0.247 **	0.209 **	−0.181 **	−0.280 **				
**Grip Strength**	0.189 **	0.186 **	0.238 **	−0.076	−0.077	−0.026	0.184 **	0.461 **	−0.240 **			
**Gait Speed**	0.164 **	0.196 **	0.181 **	−0.092 *	−0.038	−0.027	0.110 *	0.351 **	−0.241 **	0.443 **		
**IADL**	−0.073	−0.092 *	−0.335 **	0.186 **	0.100 *	−0.018	−0.295 **	−0.440 **	0.378 **	−0.259 **	−0.324 **	
**FRAIL**	−0.121 **	−0.132 **	−0.273 **	0.170 **	0.243 **	0.235 **	−0.148 **	−0.168 **	0.330 **	−0.220 **	−0.280 **	0.225 **

^a^ Data represent *r* value; ** *p* < 0.01; * *p* < 0.05; *p* values were not adjusted for location, gender or age.
